# Diabetes prediction model based on GA-XGBoost and stacking ensemble algorithm

**DOI:** 10.1371/journal.pone.0311222

**Published:** 2024-09-30

**Authors:** Wenguang Li, Yan Peng, Ke Peng

**Affiliations:** College of Computer Science and Engineering, Sichuan University of Science and Engineering, Yibin, China; Atlantic Technological University, IRELAND

## Abstract

Diabetes, as an incurable lifelong chronic disease, has profound and far-reaching effects on patients. Given this, early intervention is particularly crucial, as it can not only significantly improve the prognosis of patients but also provide valuable reference information for clinical treatment. This study selected the BRFSS (Behavioral Risk Factor Surveillance System) dataset, which is publicly available on the Kaggle platform, as the research object, aiming to provide a scientific basis for the early diagnosis and treatment of diabetes through advanced machine learning techniques. Firstly, the dataset was balanced using various sampling methods; secondly, a Stacking model based on GA-XGBoost (XGBoost model optimized by genetic algorithm) was constructed for the risk prediction of diabetes; finally, the interpretability of the model was deeply analyzed using Shapley values. The results show: (1) Random oversampling, ADASYN, SMOTE, and SMOTEENN were used for data balance processing, among which SMOTEENN showed better efficiency and effect in dealing with data imbalance. (2) The GA-XGBoost model optimized the hyperparameters of the XGBoost model through a genetic algorithm to improve the model’s predictive accuracy. Combined with the better-performing LightGBM model and random forest model, a two-layer Stacking model was constructed. This model not only outperforms single machine learning models in predictive effect but also provides a new idea and method in the field of model integration. (3) Shapley value analysis identified features that have a significant impact on the prediction of diabetes, such as age and body mass index. This analysis not only enhances the transparency of the model but also provides more precise treatment decision support for doctors and patients. In summary, this study has not only improved the accuracy of predicting the risk of diabetes by adopting advanced machine learning techniques and model integration strategies but also provided a powerful tool for the early diagnosis and personalized treatment of diabetes.

## 1 Introduction

The International Diabetes Federation (IDF) has revealed a set of concerning figures in its latest 10th edition of the Diabetes Atlas: Currently, there are approximately 537 million adults with diabetes worldwide, aged between 20 and 79, which is equivalent to one in every ten people being affected [1]. This number is expected to surge to 643 million by 2030 and further climb to 783 million by 2045. Even more worrying is that there are about 240 million undiagnosed adults with diabetes globally, which accounts for nearly half of the total number of adult patients [2]. In China, the situation is particularly severe, as the country has the highest number of undiagnosed diabetic patients, with nearly 90% of these patients concentrated in low- and middle-income countries. Especially in Africa, Southeast Asia, and the Western Pacific region, more than half of the diabetic patients have not yet been diagnosed. Diabetes not only poses a threat to individual patients but also caused the death of 6.7 million people in 2021, which is equivalent to one person losing their life every 5 seconds [3]. Economically, diabetes has led to at least $966 billion in health expenditure, a figure that has increased by 316% over the past 15 years. China ranks first in both the number of diabetic patients and undiagnosed diabetic patients, and second in the world in terms of health expenditure on diabetes, highlighting the challenges China faces with diabetes [4].

With the rapid development of big data and artificial intelligence technology, we have ushered in a new chapter of the information age. The application of artificial intelligence in the medical field is becoming more and more extensive, from intelligent guidance to medical imaging, and to auxiliary diagnosis and treatment as well as disease risk prediction. Artificial intelligence technology is continuously promoting the improvement of medical standards and reducing medical costs [5]. Machine learning, as a key branch of artificial intelligence, plays an important role in the construction of medical models and data mining. Especially in the research field of chronic diseases such as diabetes, the application of machine learning not only helps us to understand the mechanisms of disease occurrence more deeply but also provides new perspectives and tools for finding effective treatment methods. Faced with diabetes as a global health issue, the use of machine learning to carry out innovative research has become the common pursuit of the scientific and medical communities, which is of great significance for improving the prevention, diagnosis, and treatment of diabetes [6].

Against this backdrop, the early prediction of diabetes has become an urgent issue in the field of related research. Identifying high-risk individuals early on enables doctors or patients to take effective treatment measures to prevent the further progression of diabetes. In recent years, scholars at home and abroad have used data mining techniques to analyze and establish predictive models for diabetes. Applying classification algorithms to the field of disease prediction has significant practical importance for preventing diabetes and its complications [7].

This study is dedicated to exploring and predicting the risk of diabetes, employing advanced model fusion strategies to construct an accurate diabetes prediction model. This model not only has advantages in predictive accuracy but also provides an in-depth understanding and analysis of the causes of diabetes through the application of interpretability theory.

The main contributions of this paper are as follows:

1. Firstly, by comparing and contrasting various sampling techniques such as under-sampling, over-sampling, and re-sampling, the issue of data imbalance regarding the presence or absence of diabetes was effectively resolved.
2. Secondly, based on the disease prediction issue of traditional single-model algorithms, a Stacking fusion model was proposed. The main idea is to improve the overall performance of the fusion model by integrating multiple single models. The hyperparameters of the XGBoost algorithm, after data balancing, were optimized using a genetic algorithm and combined with a random forest to obtain the integrated diabetes prediction model. Experimental results show that the Stacking fusion model significantly improved its predictive accuracy compared to other single models.
3. Lastly, the interpretability component was combined with the ensemble learning model, and the SHAP framework was used to interpret the best predictive results of the ensemble learning model. There is relatively little analysis of interpretable models in this field, so this model can effectively help doctors or patients more accurately predict the risk of diabetes.

## 2 Literature review

In recent years, a considerable number of scholars have attempted to predict diabetes using data mining methods and have achieved good results. Domestically, Zheng Lin and Ni Shiwei [8] constructed a pregnancy diabetes prediction model based on SVM, which analyzed blood routine, coagulation function, and biochemical indicators in early pregnancy, achieving a prediction accuracy of 78.3% and a precision rate of 84.6%. Although the SVM model achieved good predictive results, it did not elaborate on the model’s generalization ability and its applicability to other datasets. Liu Wenbo et al. [9] applied the iterative random forest algorithm to diabetes prediction and compared it with seven other classification algorithms, finding that the iterative random forest performed best in multiple performance indicators. Although the advantages of the iterative random forest were demonstrated, the efficiency and stability of the algorithm on different data scales and dimensions were not fully considered. Wang Shijie [10] proposed the MLRStacking ensemble prediction model and the BTLMFP-growth algorithm, and verified the model’s accuracy and efficiency in diabetes prediction through experiments. Although an improved algorithm was proposed, the paper did not provide the performance of the algorithm on dealing with unbalanced datasets and possible improvement measures. Xie Nini [11] compared the advantages and disadvantages of different models and found that the Voting model performed the best, followed by KNN, SVM, and random forests. The main factors affecting diabetes were identified, and the application value of the random forest model in predicting diabetes was pointed out. Although various algorithms were compared, the impact of different feature selections on model performance was not deeply explored. Zhou Leiming et al. [12] established a diabetes prediction model based on different classifiers and found that the XGBoost model performed best with an accuracy rate of 0.8949 after comparing the classification accuracy of different models. Although a classifier model framework based on INLF was proposed, the applicability of the model to different types of diabetic patients was not analyzed. Fu Huazhen et al. [13] established a model to predict hypotension during hemodialysis in diabetic kidney disease patients, identified multiple risk factors such as age, anemia, and hypoalbuminemia, and obtained an AUC of 0.834 through the random forest model, indicating that the model had high predictive efficacy. Mei Jun and Chen Jianmin [14] analyzed the diabetes dataset through the KNN algorithm, determined the optimal K value, achieved the prediction of diabetes, and verified the effectiveness of the model through experiments. Although the two literatures established effective prediction models, they lacked application and verification of the model in actual clinical environments. Abroad, Talha Mahboob Alam et al. [15] used K-means clustering, random forest (RF), and artificial neural network (ANN) models, and concluded that ANN provided the best accuracy rate of 75.7% to assist medical professionals in treatment decisions. The study found a strong correlation between diabetes and body mass index (BMI) and blood sugar levels. However, the limitation lies in the selection of structured datasets, and non-structured data can be considered and applied to other medical fields. Deepti Sisodia and Dilip Singh Sisodia [16] used decision trees, support vector machines (SVM), and Naive Bayes on the Pima Indians Diabetes Database (PIDD) for experiments, and Naive Bayes performed best in terms of accuracy, reaching 76.30%. But it may be limited by the bias of the dataset, feature selection, and model generalization ability. Peihua Chen and Chuandi Pan [17] used Adaboost.M1 and LogitBoost algorithms, and based on clinical data, the LogitBoost model achieved an overall accuracy of 95.30% in 10-fold cross-validation and had a high rate of true positives, true negatives, false positives, and false negatives in the binary classification model. But if the original dataset is large, the conclusions may be different. In addition, the robustness of the model may be affected by missing data. Usama Ahmed et al. [18], integrated machine learning models, including support vector machines (SVM) and artificial neural networks (ANN), as well as fuzzy logic systems, and the proposed integrated ML model achieved an accuracy rate of 94.87% in diabetes prediction, higher than previously published methods. Gangani Dharmarathne et al. [19], utilized a dataset from the Pima Indians Diabetes Database to propose four classification models and an explanatory tool. Despite achieving positive outcomes, the study may face challenges due to the limitations of the dataset and the generalizability of the models. Additionally, there is room for optimization in terms of model parameter tuning. Bernice Man et al. [20], developed a 3-year diabetes risk prediction model for pre-diabetic women with a history of gestational diabetes, identifying fasting blood glucose and glycated hemoglobin as key risk factors using Cox proportional hazards regression, yet the model has limitations in generalizability and consideration of treatment adherence. Md Abdus Sahid et al. [21], utilized machine learning algorithms and feature selection techniques to develop an efficient multi-class diabetes prediction model for an Iraqi diabetes dataset, where an SVM classifier combined with a filtering method selected four features achieving an accuracy of 0.964 and an AUC of 0.99. However, confined to traditional machine learning models, these models do not offer data confidentiality, and there is potential for further improvement in parameter optimization and feature selection. Himanshu Gupta et al. [22], compared the performance of deep learning and quantum machine learning in diabetes prediction, finding that DL models outperformed QML models in predictive accuracy, although QML models still require enhancement for performance on small datasets. Praveen Talari et al. [23], developed an efficient hybrid feature selection and classification technique model by combining SMOTE and SMO algorithms for early prediction and assessment of the severity of Type 2 diabetes, achieving an accuracy of 99.07% and a runtime of 0.1 milliseconds on the Pima Indian Diabetes (PID) dataset. HuaZhong Yang et al. [24], proposed an improved ensemble learning algorithm, AWD-stacking, which combines three improved long short-term memory network models and an improved nearest neighbor propagation clustering algorithm, evaluated on the OhioT1DM dataset, achieving state-of-the-art performance, with an RMSE improvement of 27.92% and an MAE improvement of 65.32% over the best non-ensemble model, significantly enhancing predictive performance compared to existing models. Md Farhad Hossain et al. [25], employed various ML algorithms, including decision trees, random forests, support vector machines, extreme gradient boosting, K-nearest neighbors, and logistic regression, to predict Metabolic syndrome; further investigation is needed to assess the accuracy of these models as classification tools and to improve their precision.

This paper proposes a GA-XGBoost ensemble model based on the genetic algorithm optimization of XGBoost, using the Stacking fusion method, combining the advantages of multiple base learners, for constructing a diabetes prediction model. The model not only shows significant advantages in predictive accuracy but also successfully explains the key factors affecting diabetes risk through in-depth application of interpretability theory. Through this model, doctors and patients can obtain more accurate predictions of diabetes risk, thus providing strong support for early diagnosis and intervention, and helping to improve the efficiency of diabetes diagnosis and treatment and the quality of life of patients.

## 3 Material and methods

### 3.1 Technical route

**Fig 1:**
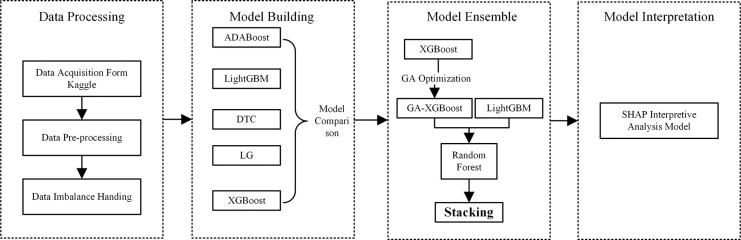
Technical approach.

This article constructs a diabetes prediction model based on the GA-XGBoost Stacking ensemble model and uses the SHAP framework for model interpretation, which is mainly divided into the following steps:

1. Obtain the Behavioral Risk Factor Surveillance System dataset from the official Kaggle website;
2. Perform data preprocessing on the original dataset, using the Pearson correlation coefficient and chi-square test for feature selection;
3. Address the issue of uneven data distribution in the dataset, including methods such as oversampling, undersampling, resampling, and hybrid sampling;
4. Construct five learning models including XGBoost, ADABoost, LightGBM, Decision Tree, and Logistic Regression for model training; Optimize the XGBoost model with various algorithms to determine the best optimization algorithm; Then use the Stacking method to integrate the optimized XGBoost and LightGBM models; Compare the performance of single models and Stacking ensemble models through experiments;
5. Use the SHAP framework to interpret the model’s predictive results. The entire process is implemented in Python, and the technical route of this study is shown in Fig 1.

### 3.2 Related theories

#### 3.2.1 XGBoost

XGBoost (eXtreme Gradient Boosting), also known as the extreme gradient boosting tree, is an efficient machine learning algorithm based on the Boosting concept and the GBDT (Gradient Boosted Decision Trees) algorithm. It has been widely applied in various fields, including fraud detection, disease diagnosis [26], sales forecasting, demand prediction, image classification, and more. XGBoost enhances the traditional GBDT by introducing regularization terms to control overfitting and by optimizing the speed and performance of the algorithm [27]. It is a strong classifier composed of multiple weak classifiers. The basic idea of the algorithm is to continuously split the tree, and after each new tree is generated, it fits the previous split. As an enhanced tree model, XGBoost optimizes the GBDT model, effectively handling the relationships between data, and has been widely applied in fields such as data mining and recommendation systems. After training the data, the scores of all the trees obtained by the model are summed up, and the result of the summation is the predicted value corresponding to the model. The XGBoost model is as shown in the formula:

$${\hat{y}}_{i}^{t}=\sum_{k=1}^{K}f_{k}(x_{i}),f_{k}\in F$$ (1)

In the formula: ${\hat{y}}_{i}^{t}$ represents the model prediction for the t-th round, *K* indicates the number of trees included in the model, *f*_*k*_ represents the quantity-related relationship between the structure *q* and the leaf weights of the k-th tree in the model, *X*_*i*_ represents the feature represented by the i-th tree in the model, and *FF* represents the space where the trees included in the model are located.

The objective function is as shown in the formula.


$$X=\sum_{i=1}^{n}l(y_{i}{\hat{y}}_{i})+\sum_{k=1}^{k}\Omega (f_{k})$$ (2)

$$\Omega (f_{k})=\gamma T+\lambda \frac{1}{2}\sum_{j=1}^{T}\omega_{j}^{2}$$ (3)

In the formula: $\sum_{i=1}^{n}l(y_{i}{\hat{y}}_{i})$ represents the training error, *T* denotes the number of terminal leaves, *γ* indicates the regularization strength, and *w* signifies the leaf weights. The algorithm iteratively updates the objective function through regularization to mitigate the influence of local optima, thus preventing the model from overfitting.

The updated objective function is:

$$\tau^{\left(t\right)}\approx \sum_{i=1}^{n}l(y_{i},{\hat{y}}^{\left(t-1\right)}+f_{t}(X_{i})+\Omega (f_{t}))$$ (4)

The function from the previous formula is expanded using a second-order Taylor expansion to find the objective function, hence the expansion is carried out at *f* = 0:

$$\tau^{\left(t\right)}\approx \sum_{i=1}^{n}\left[l(y_{i},{\hat{y}}^{\left(t-1\right)}+f_{t}(X_{i}))+\frac{1}{2}h_{i}f_{i}^{2}(X_{i})]+\Omega (f_{t}\right)$$ (5)

Summing over the loss function, thus the resulting objective function is:

$$X_{obj}=\sum_{j=1}^{T}[(\sum_{i\in I_{j}}g_{i})\omega_{j}+\frac{1}{2}(\sum_{i\in I_{j}}h_{i}+\lambda )\omega_{j}^{2}]+\lambda T$$ (6)

By applying a second-order Taylor expansion to the objective function and summing the loss function, the objective function is transformed into a quadratic equation, as shown in Formula (omitted here) and Formula (omitted here). The transformed objective function is then used to find the optimal values of *w* and the function value.


$$\omega_{j}^{*}=-\frac{\sum_{i\in I_{j}}g_{i}}{\sum_{i\in i_{J}}h_{i}+\lambda }$$ (7)

$$X_{obj}=-\frac{1}{2}\sum_{j=1}^{T}\frac{\sum_{i\in I_{j}}g_{i}}{\sum_{i\in I_{j}}h_{i}+\lambda }+\lambda T$$ (8)

#### 3.2.2 Genetic algorithm

**Fig 2:**
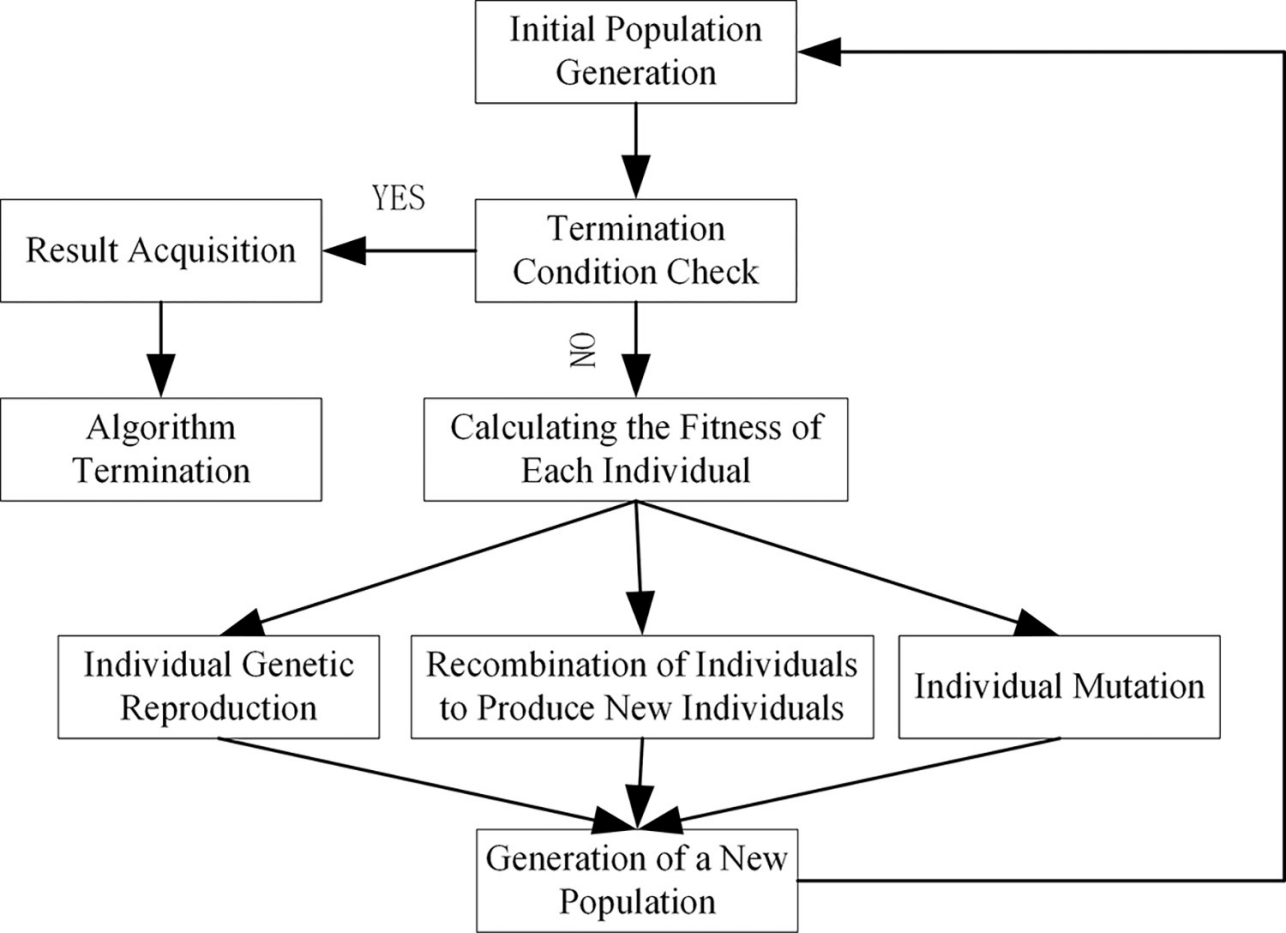
Genetic algorithm flowchart.

Genetic Algorithm is a model that simulates the mechanisms of natural selection and genetics, obtaining the optimal solution for the output by inputting the objective function and constraints [28]. Starting with an initial population, the genetic algorithm aims to generate individuals that are better adapted to the environment. It does this by using a series of operations such as random selection, crossover, and mutation, thereby evolving the entire population in the direction of a more optimal area within the search space. Through continuous reproduction and evolution, it ultimately converges in the environment to a group of individuals with strong adaptability. During the optimization process, the genetic algorithm uses probabilistic methods, where the changes in the values of the algorithm’s parameters are influenced by set probabilities, allowing it to adaptively adjust the search direction based on set probabilities without the need for fixed optimization function parameters. Due to the large dataset used in this paper, the XGBoost model indeed has many parameters, its convergence speed is relatively slow, and the hyperparameters significantly affect the prediction results. Therefore, this study adopts the parameter adjustment method of the genetic algorithm. When optimizing hyperparameters, the genetic algorithm can escape local extrema, optimize the overall optimal parameters using the individual fitness function, and continuously reduce the search area during the optimization process. Therefore, this paper proposes a GA-XGBoost model that combines the genetic algorithm with the XGBoost model, using the global search capability of the genetic algorithm to more effectively search the parameter space, improve the efficiency of optimization, and achieve exponential adjustment using AUC as the fitness function. The specific process flowchart of the genetic algorithm is shown in Fig 2.

Obtain better hyperparameters through the genetic algorithm, which are the optimal chromosome numbers obtained when the number of iterations meets the end condition. In using the genetic algorithm to optimize hyperparameters for predicting the risk of diabetes, the genetic algorithm was used to optimize and initialize the three parameters of n_estimators, learn_rate, and max_depth. The process of optimizing hyperparameters for the XGBoost model with the genetic algorithm is as follows:

1. Initialize the parameters θ;
2. Select indicators on the machine learning model;
3. Calculate the fitness function based on the selected indicators;
4. Select the optimal parameters based on fitness;
5. Perform genetics, replication, crossover, mutation, etc.;
6. Generate new parameter combinations.

#### 3.2.3 Stacking ensemble model

**Fig 3:**
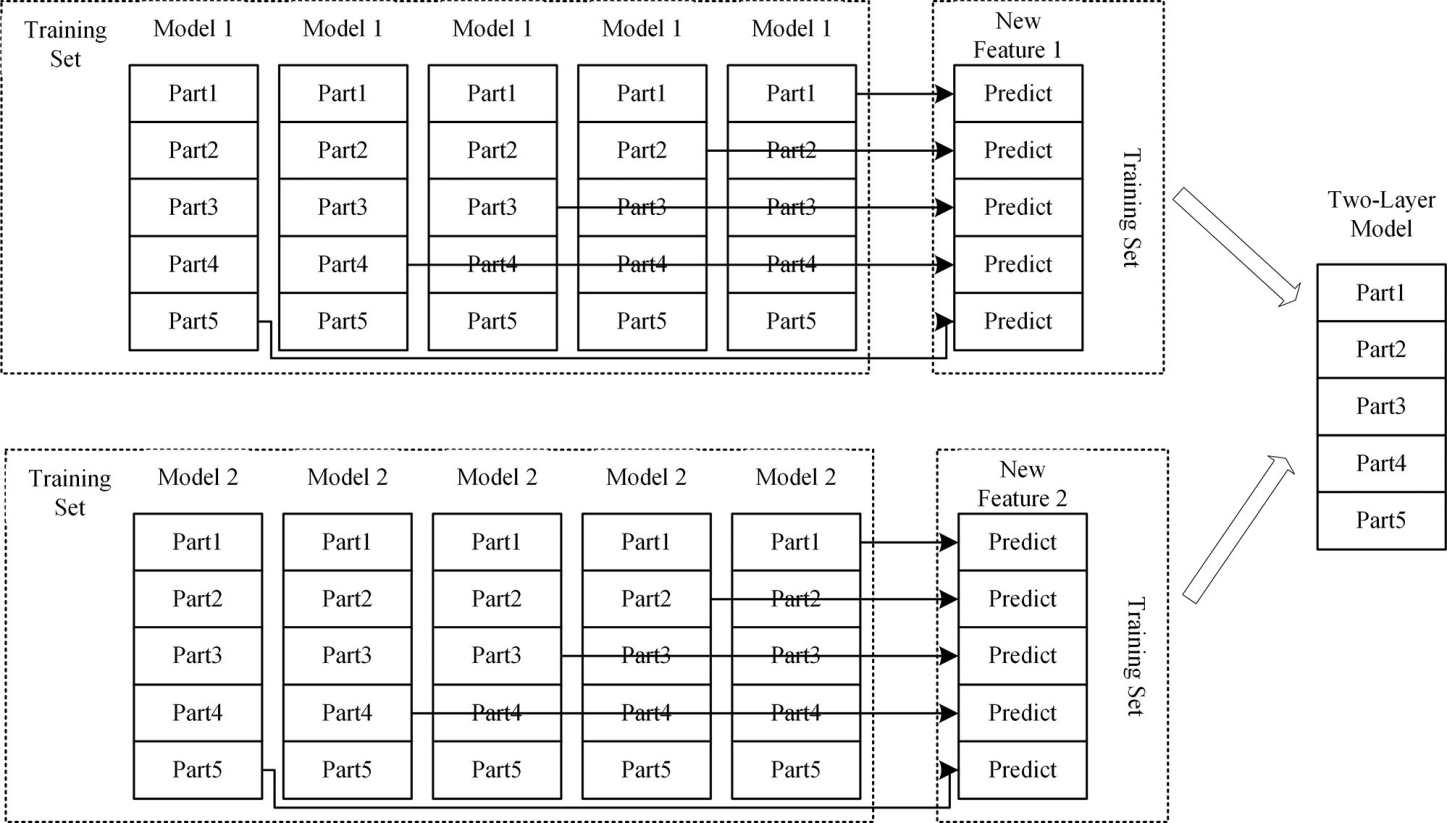
Stacking schematic diagram.

Model ensembling is an advanced machine learning technique that enhances overall predictive performance by skillfully combining multiple estimators [29]. These estimators may be homogeneous, meaning they are built based on similar algorithms or principles; or they may be heterogeneous, implying they employ different learning strategies and techniques. Among the many model ensembling techniques, Voting, Blending, and Stacking are several common methods. In this study, we will use the Stacking method to integrate Boosting models in order to achieve better predictive results. The Stacking method was first proposed by Wolpert in 1992. Fig 3 shows a schematic diagram of building a Stacking ensemble model, which evenly divides the original training set into five parts, named Part1, Part2, Part3, Part4, and Part5. In this process, we first use the data from Part2 to Part5 to train the base model 1. After training is completed, model 1 is used to predict the Part1 that did not participate in the training, and the obtained prediction results constitute one-fifth of the new feature 1. Following this method, we continue to use the data from Part1, Part3, Part4, and Part5 to train model 1 and predict Part2, and so on, ultimately generating a complete new feature 1. The same method is applied to the base model 2, and through such operations, we can create an extended training set that includes two new features. This new training set is then used to train the second-layer model, which is the upper layer of the Stacking model. For the treatment of the validation set, we adopt a similar strategy. First, according to the division of the training set, we generate a dataset for the validation set that includes two new features. Then, this extended validation set is input into the well-trained second-layer model. In this way, we finally obtain the predictive output of the Stacking ensemble model. This Stacking method not only enhances the model’s generalization ability but also improves the overall model’s accuracy and robustness by effectively combining the predictions of multiple base models.

In the typical structure of Stacking, the model is divided into two levels: The first level consists of multiple base models, which are usually complex models with high fitting degrees, such as XGBoost, LightGBM, SVM, etc. Their main task is to learn from the training data and capture the characteristics of the data as much as possible. The second level is composed of one or more relatively simple models, such as logistic regression, Lasso regression, and other generalized linear models. The role of these models is to integrate the predictive results of all base models and generate the final predictive output. Since the base models at the first level may have a high degree of nonlinearity and complex feature extraction capabilities, they learn specific patterns of the training data to varying degrees. This diversity, while helpful in improving the model’s expressive power, can also lead to the risk of overfitting, especially when there is a high degree of similarity between the base models. To reduce this risk, the choice of models for the second level tends to be simpler, in hopes of balancing the complexity of the first level through their regularizing effect, thus avoiding overfitting. This paper, through a carefully designed Stacking fusion strategy, not only makes full use of the advantages of each base model but also improves the model’s generalization ability and robustness through the integration of the second-level model.

### 3.3 Experimental data and preprocessing

#### 3.3.1 Data source

The research in this paper is based on the 2021 BRFSS (Behavioral Risk Factor Surveillance System Dataset) provided by the Kaggle platform, which is a leading health-related telephone survey system in the United States. The system is dedicated to collecting state-level data on health-related risk behaviors, chronic health conditions, and the use of preventive services among U.S. residents. The dataset is vast, containing 438,693 records and covering 303 different feature variables. Faced with such a large dataset and numerous features, in our experiments for the diabetes prediction model, we carefully selected 25 features closely related to the research objectives for in-depth analysis. Through this strategy, we aim to simplify the model training process while ensuring that the selected features can effectively capture information related to the prediction of diabetes risk. This approach not only improves the efficiency of model training but also helps to enhance the model’s predictive accuracy and interpretability. The following is the URL for the dataset: https://www.kaggle.com/datasets/dariushbahrami/cdc-brfss-survey-2021/data

#### 3.3.2 Data preprocessing

Data preprocessing is an essential step in machine learning and data analysis, referring to the collection, cleaning, transformation, and scaling of raw data to better adapt it for the application of machine learning algorithms. The main purpose of data preprocessing is to extract useful information from the raw data, eliminate irrelevant or invalid information, overcome noise interference, reduce the complexity of data dimensions, and thereby improve the performance and universality of the model. This paper first deals with missing values by deleting them, leaving 3,303,545 records in the dataset after deletion; then, using the BRFSS codebook as a reference, each feature is modified, and values are cleaned to make them more suitable for algorithm implementation; finally, columns are renamed to improve clarity and readability. A version with diabetes as the target variable is saved. It is clean but unbalanced. A binary dataset of diabetes and non-diabetes is created, which is the dataset used for subsequent experiments, as shown in Table 1.

**Table 1:** Data set presentation.

No.	Column	Non-Null Count	Dtype	No.	Column	Non-Null Count	Dtype
0	Diabetes_binary	234951 non-null	float64	13	EatVegetables	234951 non-null	int64
1	BMI	234951 non-null	float64	14	GenHlth	234951 non-null	float64
2	Overweight	234951 non-null	int64	15	PhyHlth	234951 non-null	float64
3	HighBP	234951 non-null	int64	16	MentHlth	234951 non-null	float64
4	HighChol	234951 non-null	float64	17	DiffWalk	234951 non-null	float64
5	CholCheck	234951 non-null	int64	18	HlthCareAccess	234951 non-null	int64
6	KidneyProbl	234951 non-null	float64	19	NoDocCost	234951 non-null	float64
7	Smoker	234951 non-null	float64	20	RegCheckup	234951 non-null	float64
8	Alcohlic	234951 non-null	int64	21	SEX	234951 non-null	int64
9	HadStroke	234951 non-null	float64	22	Age	234951 non-null	int64
10	HeartProbl	234951 non-null	float64	23	Education	234951 non-null	float64
11	PhyActivity	234951 non-null	int64	24	Income	234951 non-null	float64
12	EatFruits	234951 non-null	int64				

#### 3.3.3 Feature selection

**Fig 4:**
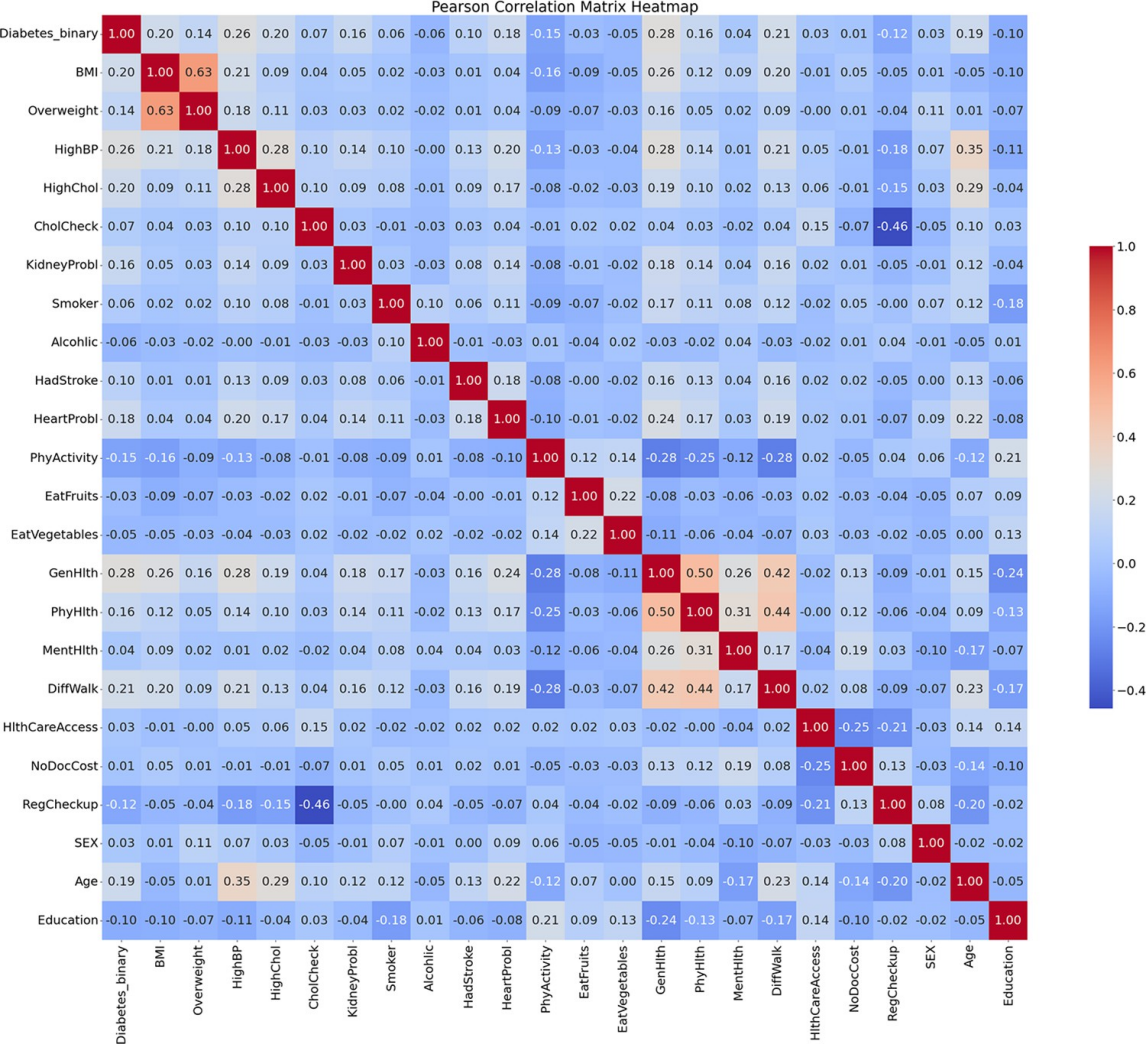
Pearson correlation coefficient matrix plot.

During an in-depth analysis of the dataset, we found that the variables in the dataset are primarily continuous. To explore the relationships between these variables, we employed the Pearson correlation coefficient, a statistical tool. The Pearson correlation coefficient is an important measure for assessing the strength and direction of the linear relationship between two continuous variables, with values ranging from -1 to 1. Specifically, when the absolute value of the Pearson correlation coefficient reaches between 0.8 and 1.0, it indicates a very strong correlation between the two variables; if the value is between 0.6 and 0.8, it suggests a moderately strong correlation; if between 0.4 and 0.6, it indicates a moderate correlation; if between 0.2 and 0.4, it is considered a weak correlation; and when the absolute value of the correlation coefficient is less than 0.2, it is generally believed that there is either a very weak relationship or no linear relationship between the two variables. In this paper, we visually presented the correlations between the variables in the dataset by constructing a Pearson correlation coefficient matrix plot (as shown in Fig 4).

The scale on the right side of the graph illustrates the depth of color corresponding to different correlation coefficients. From the graph, it can be observed that there is a high correlation between BMI and overweight, which is 0.63, indicating the presence of strong multicollinearity. When conducting feature engineering, it may be advisable to remove one of the two variables to prevent overfitting caused by multicollinearity.

**Fig 5:**
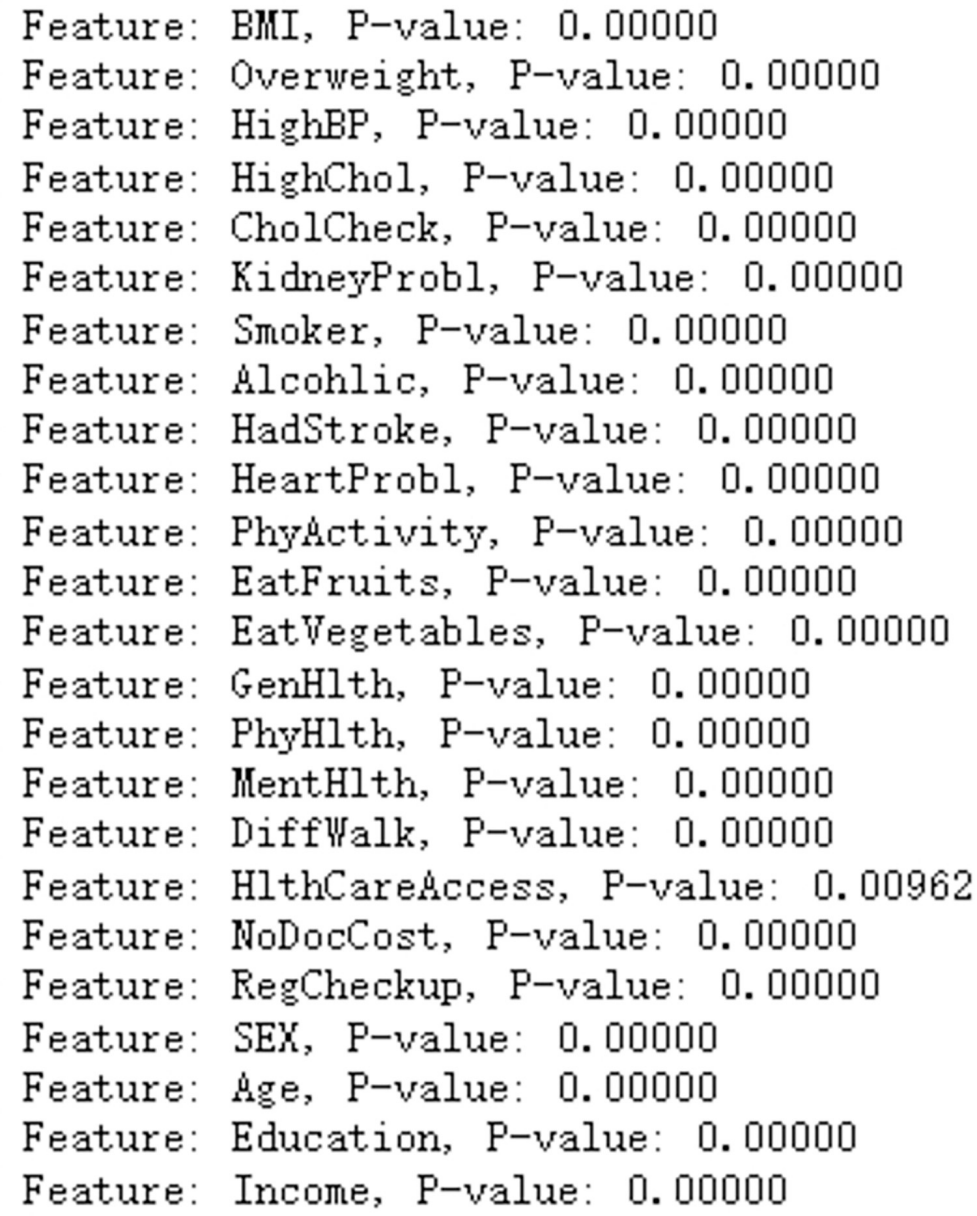
Partial results of the chi-square test.

Next, the chi-square test is employed. The chi-square test is a statistical method used to analyze whether there is a statistically significant association between categorical variables. During the chi-square test, the P-value is the indicator used to determine whether the difference between the observed sample data and the assumed distribution (usually a random distribution) is significant. The smaller the P-value, the greater the difference between the observed sample data and the assumed distribution, thus increasing the likelihood of rejecting the null hypothesis. Partial results of the chi-square test are shown in Fig 5. By calculating the P-value for each feature, the experiment shows that all feature P-values are less than 0.05, which is the most common level of significance in scientific research. It indicates that if the P-value is less than 0.05, we have sufficient evidence to reject the null hypothesis and believe that there is a significant association between the categorical variables. Combining both methods, all features are retained for model training.

### 3.4 Experimental environment and evaluation metrics

In the experiment, tools such as XGBoost, ADABoost, LightGBM, Imbalance, and Scikit-learn were utilized, employing the Jupyter Notebook platform and writing algorithms in Python 3.6 language.

Regarding diabetes prevalence, due to the imbalance between the diseased and non-diseased data in the dataset, we selected learning metrics for evaluating diabetes prevalence, focusing the evaluation metrics on the diseased. The accuracy, recall, precision, and F1 score metrics were obtained from the confusion matrix [30], as shown in Table 2. TP represents true positives (actual and predicted disease); FN represents false negatives (actual disease, but predicted as non-diseased); FP represents false positives (actual non-diseased, but predicted as diseased); TN represents true negatives (actual and predicted non-diseased).

**Table 2:** Confusion matrix.

Actual value	Predictive value	
	Diabetes	Not Diabetes
Diabetes	TP	FN
Not Diabetes	FP	TN

In the prediction issue of diabetes prevalence, the evaluation metrics based on the confusion matrix are accuracy, precision, recall, and the F1 score [31]. The specific formulas are as follows:

$$Accuracy=\frac{TP+TN}{TP+TN+FP+FN}$$ (9)

$$\mathrm{Pre}cision=\frac{\mathrm{TP}}{\mathrm{TP}+\mathrm{FP}}$$ (10)

$$\mathrm{Recall}=\frac{\mathrm{TP}}{\mathrm{TP}+\mathrm{FN}}$$ (11)

$$F1-score=\frac{2\times Precision\times Recall}{Precision+Recall}$$ (12)

Accuracy refers to the proportion of correct predictions made by the classifier. In this study, precision is the proportion of samples predicted as diseased that are correctly predicted, reflecting the effectiveness of the prediction for diseased samples. Recall is the proportion of samples that are actually diseased and are correctly predicted as diseased, reflecting the correct rate of identifying diseased samples. Looking at the formula for the F1-score, the F1-score is the geometric mean of precision and recall, balancing both metrics and serving as an alternative to them. The higher the F1-score, the better the model’s performance, and vice versa. Additionally, the ROC curve, or the receiver operating characteristic curve, is also an evaluation metric derived from the confusion matrix. The horizontal axis represents the false positive rate, and the vertical axis represents the true positive rate. The formulas for calculating the TP rate and FP rate are as follows:

$$\mathrm{TPR}=\frac{\mathrm{TP}}{\mathrm{TP}+\mathrm{FN}}$$ (13)

$$\mathrm{FPR}=\frac{\mathrm{FP}}{\mathrm{FP}+\mathrm{TN}}$$ (14)

The AUC value is the area under the ROC curve, which is plotted with the TPR (True Positive Rate) on the vertical axis and the FPR (False Positive Rate) on the horizontal axis. The AUC value is used for the overall evaluation of the diabetes prediction model; the closer the AUC value is to 1, the better the performance of the diabetes prediction model. An AUC of 0.5 indicates that the model has no effect; an AUC less than 0.5 suggests that there may be errors in the data processing.

## 4 Results

### 4.1 Handling of imbalanced datasets

**Fig 6:**
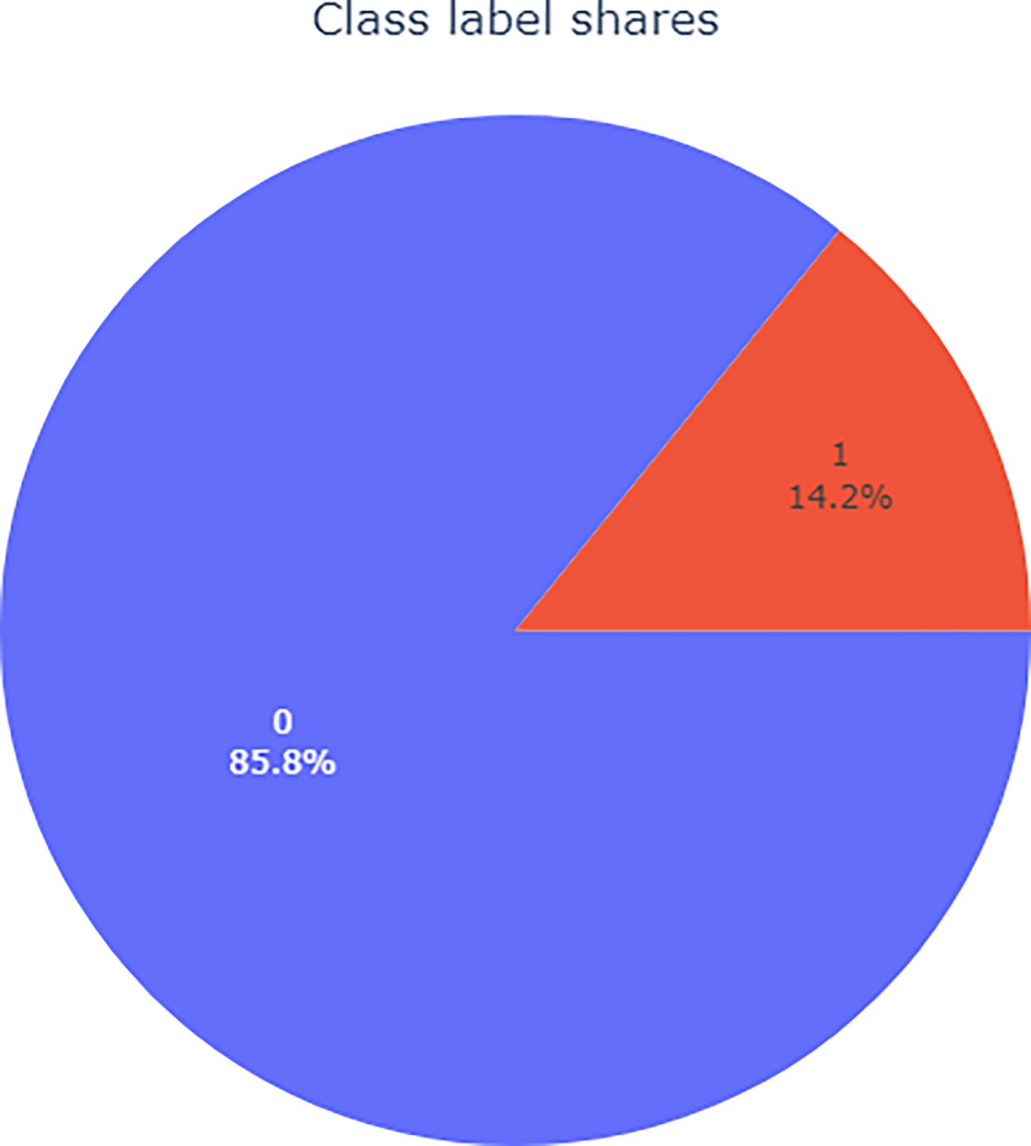
Demonstration of data imbalance.

Analyzing the distribution of diabetes cases and non-cases in Fig 6, it is evident that the proportion of individuals with diabetes is relatively low at 14.2%, with a ratio of about 6:1 between those with and without the disease. The dataset is highly imbalanced, making it easy for cases with diabetes to be predicted as the majority class, leading to poor predictive results. Using the XGBoost model for prediction, although the accuracy can reach 86.52%, the recall rate is only 17.34%, and the AUC value is 0.8282, indicating that the model’s classification effect is weak and it tends to predict cases with diabetes as the majority class, resulting in poor predictive outcomes. To address the issue of data imbalance, this paper employs various sampling algorithms, altering the data distribution to achieve balanced processing. In the experiments, oversampling algorithms such as RandomOverSampler, ADASYN, SMOTE, and the hybrid sampling method SMOTEENN were used. The data processed by different sampling algorithms were input into the XGBoost model for training, and the results were evaluated using multiple evaluation metrics to ultimately select the best-performing algorithm for handling data imbalance. Table 3 shows a comparison of the metrics for different sampling algorithms in the XGBoost model.

**Table 3:** Performance comparison of different adoption algorithms in XGBoost model.

Sampling algorithm	AUC	Accuracy	Precision	Recall	F1-score
No processing	0.8282	0.8652	0.5914	0.1734	0.2682
RandomOverSampler	0.8369	0.7576	0.7343	0.8076	0.7692
ADASYN	0.9632	0.8981	0.9448	0.8466	0.8930
SMOTE	0.9627	0.8984	0.9423	0.8488	0.8932
SMOTEENN	**0.9835**	**0.9325**	**0.9441**	**0.9473**	**0.9457**

The SMOTE algorithm augments the quantity of minority class samples by synthetically generating new instances, primarily through the method of linear interpolation of the minority class samples to create new data points [32]. On the other hand, the ADASYN algorithm creates a new minority class instance by interpolating a certain proportion of the minority class instances. In contrast to SMOTE, the ADASYN approach more heavily emphasizes the challenge of distinguishing minority class instances, enabling the generation of a greater number of minority class instances around the positive class instances. The SMOTEENN algorithm is a hybrid sampling technique that combines the SMOTE algorithm with the ENN (Edited Nearest Neighbors) algorithm. It initially employs the SMOTE method to augment the minority class instances through linear interpolation and subsequently utilizes the ENN algorithm to eliminate majority class instances. The ENN algorithm removes noise instances that are dissimilar from the majority of their K nearest neighbors.

After conducting an in-depth analysis of the experimental results, we reached some key findings. The experiment clearly revealed the significant advantage of sampling algorithms over the original untreated data samples in terms of evaluation metrics. The improvement in these metrics indicates that using sampling algorithms to balance the dataset is an effective means of constructing a diabetes prediction model. After a thorough comparison of the four different sampling algorithms, we found that the hybrid sampling method, SMOTEENN, performed exceptionally well in all key performance indicators. Specifically, the SMOTEENN algorithm achieved an AUC of 98.71%, an accuracy of 94.18%, a recall of 95.51%, a precision of 95.08%, and an F1-score of 95.30%, all of which are superior to the other three sampling algorithms.

Based on these results, we can conclude that the SMOTEENN algorithm is the best choice for balancing the diabetes dataset. It not only has advantages in predictive accuracy but also shows high efficiency in running time, which is of great significance for model deployment and real-time prediction in practical applications. Therefore, using the SMOTEENN algorithm to optimize the data preprocessing phase of the diabetes prediction model is expected to achieve higher predictive performance and clinical application value.

### 4.2 Comparison of single model experimental results

This paper combines the SMOTEENN algorithm with machine learning classification algorithms. In addition to selecting three ensemble classification models, XGBoost, ADABoost, and LightGBM, for performance comparison, traditional algorithm models such as Decision Tree and Logistic Regression are also compared for performance, as shown in Table 4 and Fig 5.

**Table 4:** Comparison of results from various classification models.

Classifier	AUC	Accuracy	Precision	Recall	F1-score
XGBoost	**0.9835**	**0.9325**	**0.9441**	**0.9473**	**0.9457**
ADABoost	0.9685	0.9068	0.9163	0.9351	0.9256
LightGBM	0.9803	0.9263	0.9379	0.9436	0.9408
DecisionTree	0.9346	0.8727	0.8913	0.9049	0.8981
LogisticRegression	0.9228	0.8550	0.8682	0.9032	0.8854

**Fig 7:**
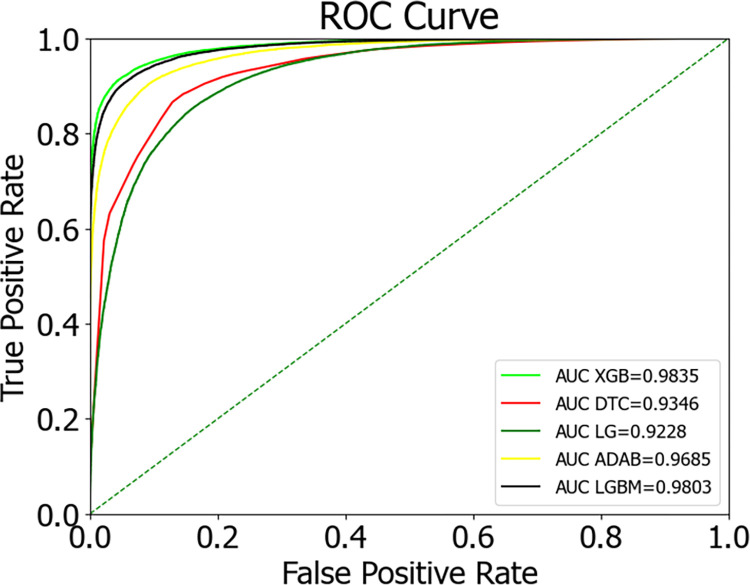
Comparison of ROC curves for various classification models.

Comparing the results from Table 4 and Fig 7, it is evident that in the study of diabetes prediction models, the XGBoost model stands out for its exceptional performance. This model excels in key performance indicators: the accuracy rate reaches 93.25%, the precision is as high as 94.41%, the recall rate is 94.73%, the F1 score is 94.57%, and the AUC (Area Under the ROC Curve) even reaches 98.35, which is the most outstanding among the five models. The excellent performance of these indicators proves the efficiency and reliability of XGBoost in predicting diabetes. A notable feature of the XGBoost algorithm is its efficient handling of input data. The algorithm uses a block structure to store input data, which significantly reduces the amount of computation during prediction, thereby speeding up the prediction process [33]. This optimization not only improves the operational efficiency of the model but also makes XGBoost more adept at handling large-scale datasets. In addition, the XGBoost algorithm approximates the objective function through a second-order Taylor expansion and introduces regularization terms, effectively controlling the complexity of the model and thus avoiding the risk of overfitting. This regularization mechanism is key to the robustness of the XGBoost algorithm, ensuring that the model maintains high generalization across different datasets. Compared to other classification algorithms, XGBoost shows higher stability and accuracy. Whether it is the convergence speed during the training process or the accuracy in predicting new data, XGBoost has demonstrated its superiority. The excellent performance of the XGBoost model in the diabetes prediction model is not only reflected in its high accuracy and high AUC value but also in its efficient data processing capability, robust model performance, and excellent generalization ability.

### 4.3 XGBoost model hyperparameter tuning

Hyperparameter tuning plays a crucial role in the domain of machine learning, with its primary goal being to identify the best set of parameters to enhance the model’s predictive capabilities and generalization performance. Among the various hyperparameter tuning techniques, grid search, random search, and Bayesian optimization are some of the most widely used methods.

Grid search is an exhaustive search strategy that divides the potential values of each parameter into a series of discrete points, creating a multi-dimensional grid. The algorithm then evaluates each parameter combination on this grid by training the model and testing its performance to ultimately select the optimal combination. While comprehensive, this method can be computationally expensive, particularly when dealing with a high number of parameter dimensions. Due to the large volume of data in this study, multiple trials were conducted for the grid search parameters, resulting in the following values: ’max_depth’: [3, 4, 5, 6], ’learning_rate’: [0.01, 0.05, 0.1], ’n_estimators’: [100, 200].

Random search is a more efficient heuristic method that samples randomly within the parameter space rather than systematically exploring all possible combinations. By making fewer attempts, this method can uncover parameter configurations with good performance, reducing search costs and circumventing the dimensionality issues associated with grid search. In this study, the parameters for random search were set as follows: ’max_depth’: [3, 4, 5, 6, 7, 8], ’learning_rate’: [0.01, 0.1, 0.3], ’n_estimators’: [100, 200, 300].

Bayesian optimization is an intelligent search strategy that utilizes probabilistic models to predict parameter performance and guide the search process. It constructs a Gaussian process model for the objective function, directing the search towards the most promising regions to find a parameter set close to the optimum in a smaller number of iterations. In this study, the parameters for Bayesian search were set as: ’max_depth’: (3, 10), ’learning_rate’: (0.01, 0.3), ’n_estimators’: (100, 500).

**Fig 8:**
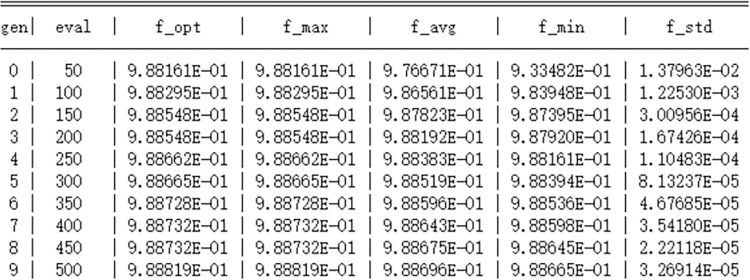
Genetic algorithm iteration data chart.

**Fig 9:**
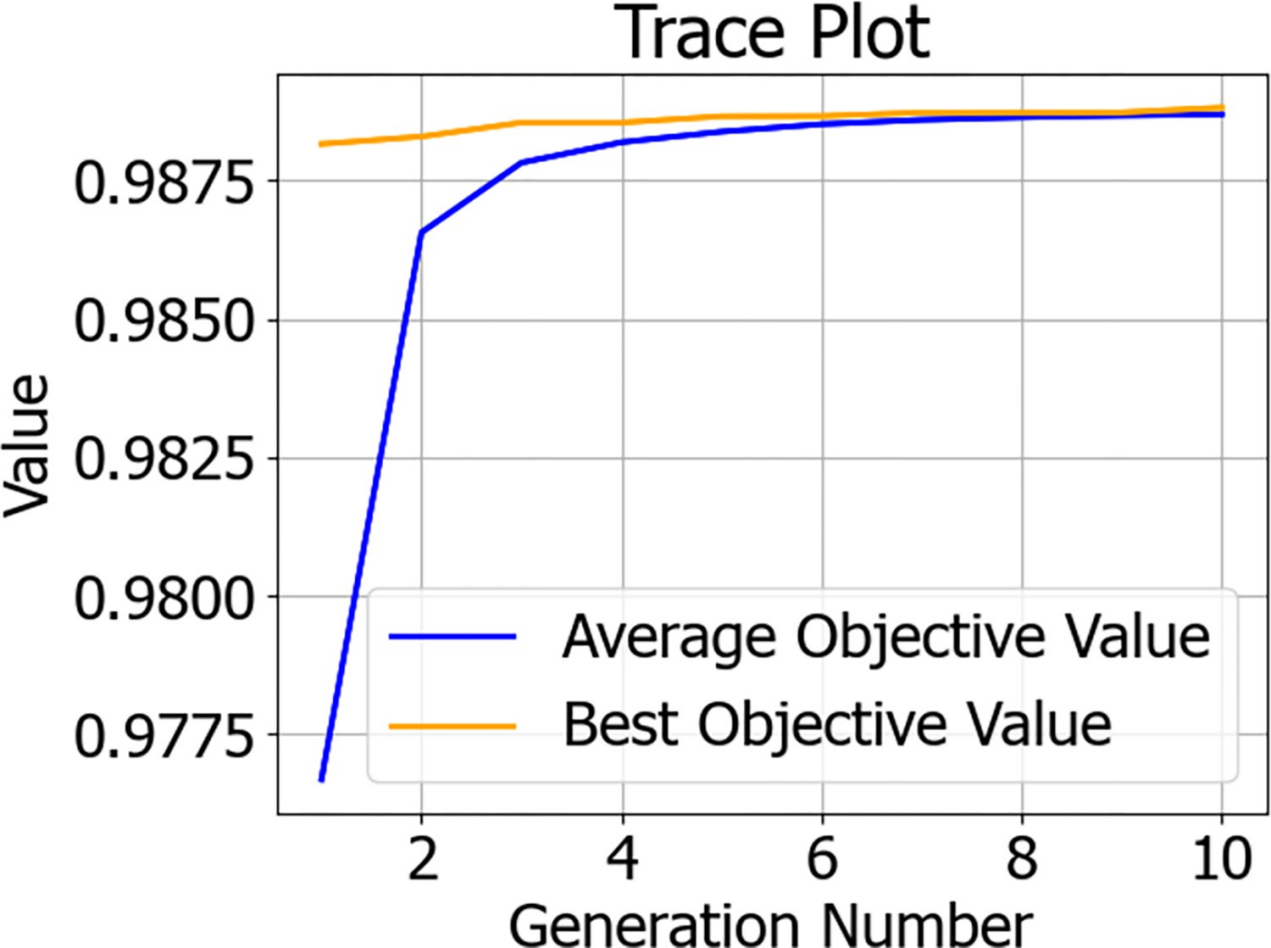
The Search process of the genetic algorithm.

In addition to these traditional methods, this paper also introduces a genetic algorithm for hyperparameter tuning, which is a meta-heuristic search algorithm that simulates the mechanisms of natural selection and genetics. The genetic algorithm iteratively optimizes the solution set by simulating the biological evolutionary process, using operations such as selection, crossover, and mutation, effectively escaping local optima and approaching the global optimum. In the hyperparameter tuning of the XGBoost model, the genetic algorithm assesses the performance of parameter combinations by defining a fitness function and continuously optimizes the search space during the iteration process, aiming to achieve better model performance. Based on the characteristics of the genetic algorithm and the range of XGBoost parameters, the genetic algorithm tuning parameters are set as follows: a population size of 50, a maximum of 10 evolutionary generations, logging information every 1 iteration, a threshold value of 1e-6 for determining when single-objective optimization is stuck, and a maximum limit of 10 for the evolution stagnation counter. After 10 iterations, the optimal individual’s objective function value in the population converges with the average objective function value of the population individuals, and the optimal fitness function AUC of 98.88% is searched. The approximate optimal solution for GA-XGBoost is n_estimators = 300, learning_rate = 0.18, max_depth = 10. The iterative data of the genetic algorithm are shown in Fig 8, and the search process of the genetic algorithm is shown in Fig 9.

The forecast outcomes of the XGBoost model, post hyperparameter optimization, are contrasted as illustrated in Table 5.

**Table 5:** Comparison of XGBoost hyperparameter tuning optimization results.

Hyperparameter Optimization Techniques	AUC	Accuracy	Precision	Recall	F1-score
Default Parameters	0.9835	0.9325	0.9441	0.9473	0.9457
Random Search	0.9880	0.9445	0.9618	0.9480	0.9549
Grid Search	0.9863	0.9395	0.9553	0.9467	0.9509
BayesSearch	0.9884	0.9482	0.9631	0.9529	0.9580
Genetic Algorithm	**0.9888**	**0.9482**	**0.9646**	0.9513	0.9579

By comparing the predictive results, we found that the XGBoost model, optimized by the genetic algorithm, demonstrated a significant performance improvement in predicting diabetes risk. Specifically, the model achieved an AUC value of 98.88%, an accuracy (Accuracy) of 94.82%, and a precision (Precision) of 96.46%. Although the recall rate (Recall) and the F1 score (F1-Score) are relatively lower, the overall performance has surpassed that of the other three traditional optimization algorithms. These results indicate that the genetic algorithm has a distinct advantage in optimizing the XGBoost model for predicting the risk of diabetes.

### 4.4 Model fusion

Through the aforementioned hyperparameter optimization of the model, the optimized GA-XGBoost model has demonstrated excellent performance, leading us to decide to incorporate it as part of the Stacking model fusion strategy. Additionally, the LightGBM model has shown remarkable performance. Known for its robustness and effectiveness in handling various datasets, the LightGBM model [34] naturally became another base classifier in our Stacking model. After establishing the base classifiers, the choice of the meta-classifier was considered. In the Stacking model, the role of the meta-classifier is to synthesize the predictive outcomes of the base classifiers to generate the final prediction output. Although there are many complex models to choose from, based on previous research and practice, we have found that simple models often provide satisfactory performance in the role of the meta-classifier. Therefore, we selected the random forest as the meta-classifier, favored for its powerful ensemble learning capabilities and its tolerance for the diversity of base classifiers. To enhance the predictive accuracy of the meta-model and thus enable it to more accurately assess the performance of other models, while preventing overfitting in the random forest itself and ensuring its generalization ability, we employed random search for hyperparameter optimization of the random forest, ensuring that it can provide accurate, stable, and efficient assessments when serving as the meta-model.

**Fig 10:**
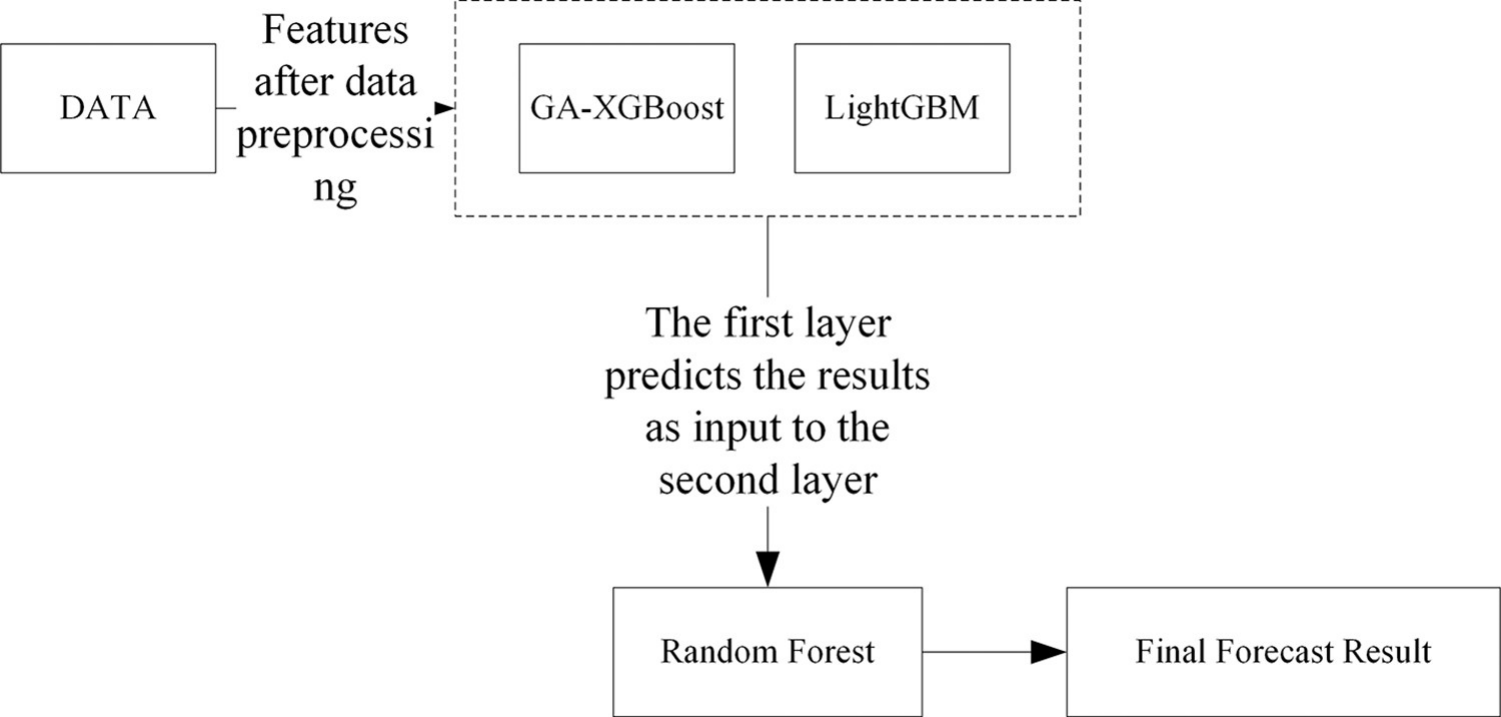
Flowchart of stacking model integration.

**Fig 11:**
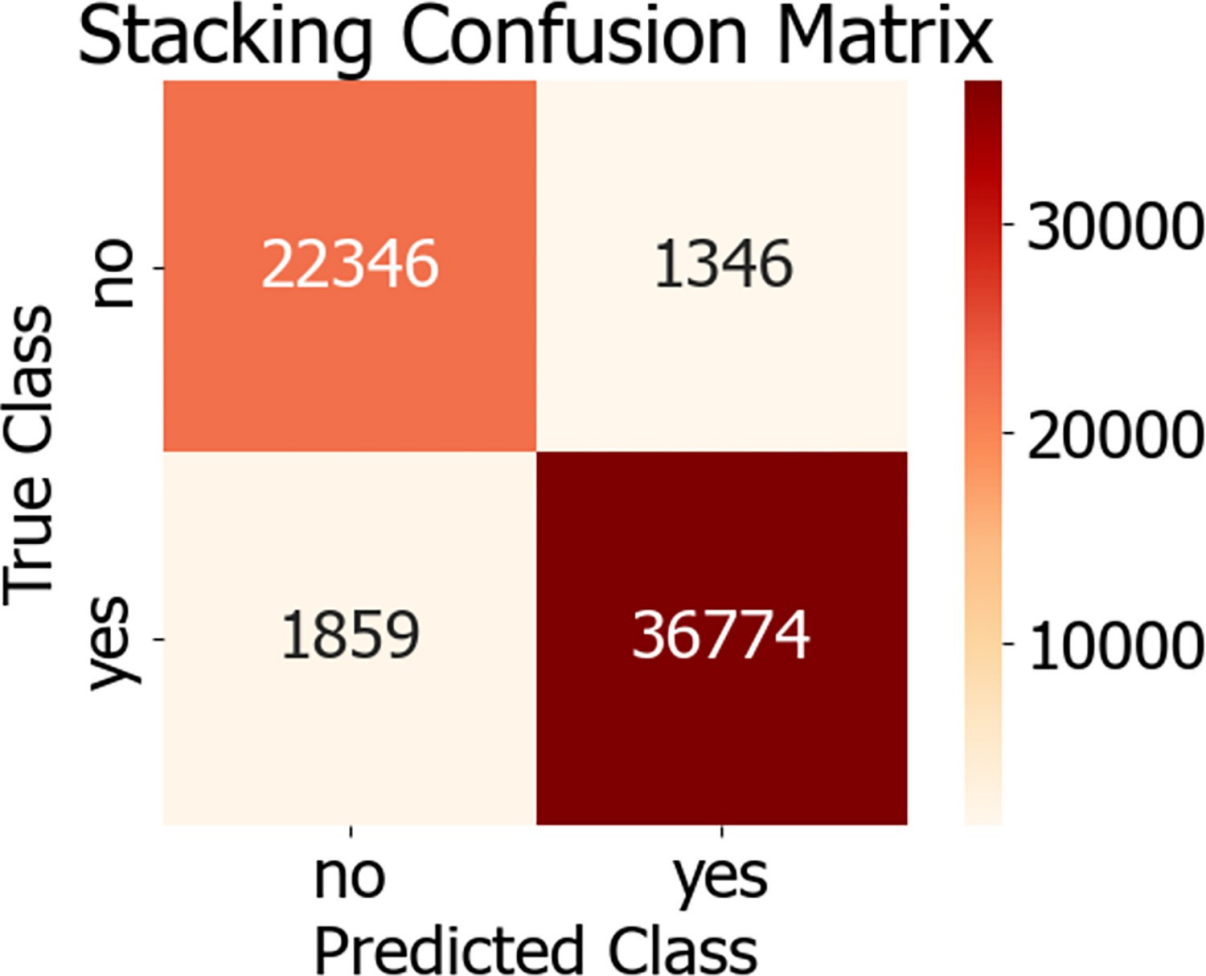
Stacking confusion matrix.

**Fig 12:**
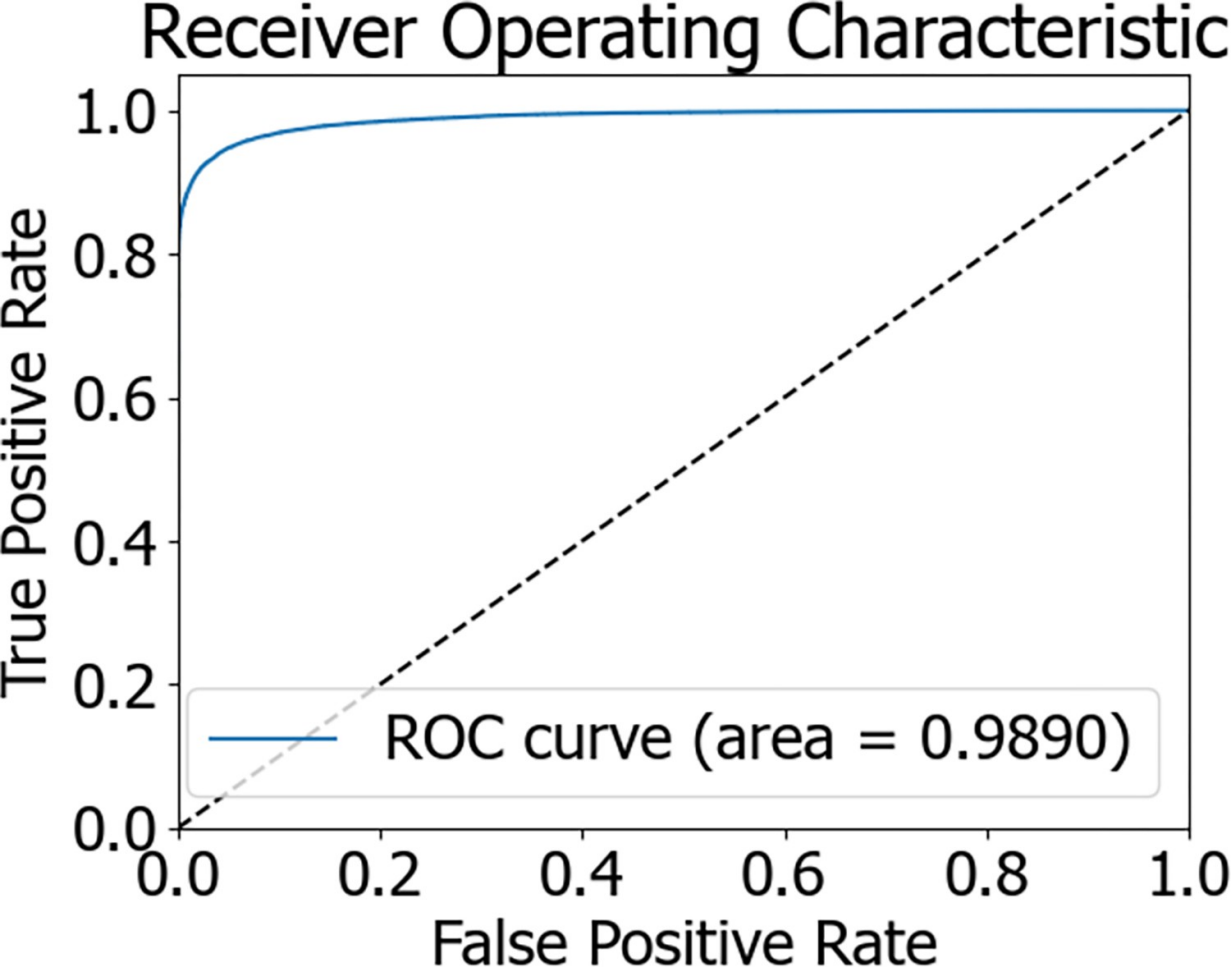
Stacking ROC curve chart.

The flowchart of the Stacking model integration is depicted in Fig 10. During the experiment, initially, 80% of the dataset was allocated as the training set, with the remaining 20% serving as the test set. The training set was trained using a 5-fold cross-validation approach. Within this set, every 4 parts of the data were designated as the training subset, while the other part was termed the validation subset. Training was conducted on the 4 training subsets, and predictions were made for both the validation subset and the original test samples. In the end, each model received 5 sets of fitting values from the test data and corresponding fitting values from the training data. The obtained predictions were averaged, which is akin to obtaining two sets of predictions from two models. Ultimately, the outputs from the base classifiers were fed into the meta-classifier, a logistic regression model, which underwent training and testing to achieve the predictive outcome of the Stacking model fusion. The predictive results of the Stacking model are presented in Table 6. The AUC value of the Stacking model’s final prediction reached 98.90%, signifying an enhanced predictive performance. The confusion matrix and ROC curve of the model are illustrated in Figs 11 and 12, respectively.

**Table 6:** Prediction results of the stacking model.

Classifier	AUC	Accuracy	Precision	Recall	F1-score
Stacking	0.9890	0.9486	0.9647	0.9519	0.9582

### 4.5 Model comparison analysis

**Fig 13:**
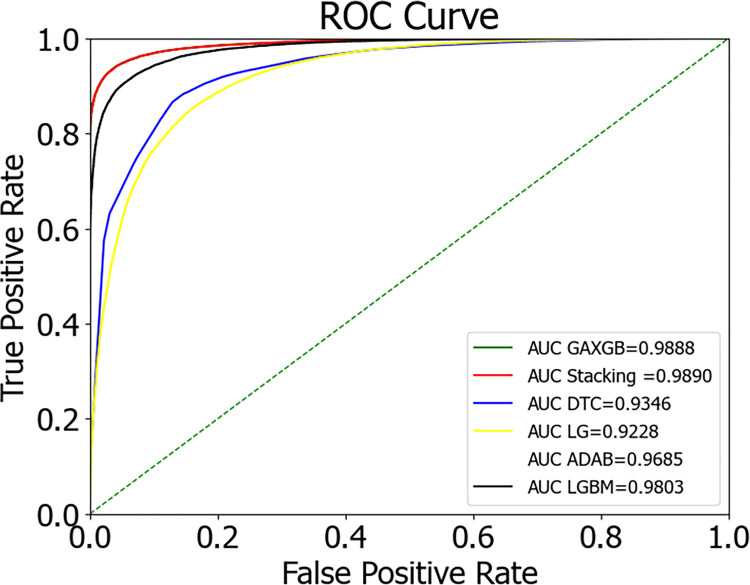
Comparison chart of ROC curves for multiple models.

Following the balanced processing of the diabetes patient dataset, we developed six distinct machine learning models, encompassing AdaBoost, LightGBM, Decision Tree, Logistic Regression, XGBoost optimized by Genetic Algorithm (GA-XGBoost), and the Stacking ensemble model. A comparison of the ROC curves for these models is presented in Fig 13, while a comparison of the model predictive outcomes is detailed in Table 7. Notably, the Stacking ensemble model demonstrated superior performance across various evaluation metrics, including an AUC of 98.90%, accuracy of 94.86%, precision of 96.47%, recall rate of 95.19%, and an F1-score of 95.82%—representing the highest levels among the models under comparison. The experimental findings suggest that the Stacking ensemble model is the most efficacious for predicting diabetes risk, which carries significant implications for patient treatment plans and offers invaluable diagnostic insights to physicians.

**Table 7:** Comparison of prediction results from multiple models.

Classifier	AUC	Accuracy	Precision	Recall	F1-score
XGBoost	0.9871	0.9418	0.9551	0.9508	0.9530
ADABoost	0.9783	0.9221	0.9293	0.9466	0.9378
LightGBM	0.9711	0.9137	0.9197	0.9432	0.9313
DecisionTree	0.9455	0.8882	0.9193	0.8988	0.9089
LogisticGegression	0.9254	0.8586	0.8715	0.9057	0.8883
GA-XGBoost	0.9888	0.9482	0.9646	0.9513	0.9579
Stacking	**0.9890**	**0.9486**	**0.9647**	**0.9519**	**0.9582**

### 4.6 SHAP interpretation model

**Fig 14:**
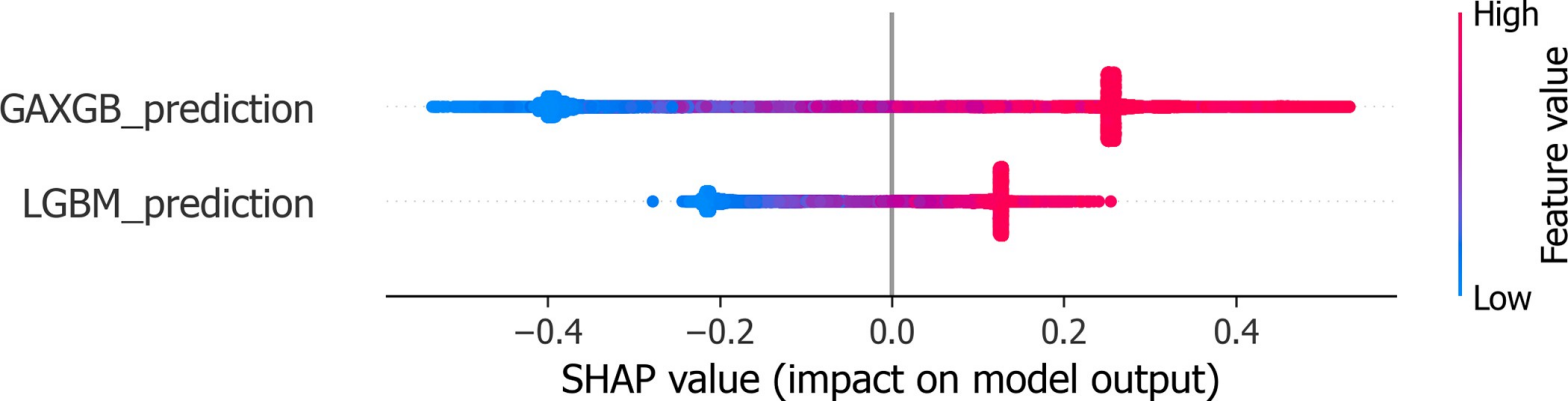
SHAP of stacking.

Due to the model functioning as a black box, it is infeasible to offer a rational explanation for the model’s predictive outcomes. SHAP (SHapley Additive exPlanations) is a machine learning interpretation method grounded in game theory, providing a model-agnostic approach to assess the contribution of individual features to the model’s predictions [35]. It can aggregate these individual contributions into an explanation for the model’s overall behavior. For opaque models, SHAP can unveil the manner in which input features influence the model’s forecasts. This is accomplished by computing the marginal contribution of each feature, thereby enabling an understanding of each feature’s positive or negative effect on the ultimate prediction. At the same time, in medical diagnosis, the explainability of models is crucial for gaining the trust of doctors and patients. The SHAP algorithm can provide explanations for each prediction outcome, demonstrating the specific impact of different clinical features on the model’s output. By analyzing SHAP values, clinical features that have the greatest impact on disease diagnosis or patient prognosis can be identified, which helps medical experts focus on the most important biomarkers. In medical research, using the SHAP algorithm can verify whether the model relies on known, biologically meaningful risk factors, thereby increasing the model’s credibility and guiding model improvements [36]. Consequently, this study incorporates the SHAP framework to analyze the model’s predictive outcomes, as depicted in Fig 14, which illustrates the SHAP feature density scatter plot for the Stacking model. The input features are the predictions when GA-XGBoost and LightGBM are utilized as base learners. The features are ordered, with the horizontal axis at the base indicating the model’s SHAP values, sorted by the average magnitude of the SHAP values. Dots symbolize samples, with more red indicating higher feature values and more blue indicating lower values. The sign of the SHAP values indicates the positive or negative contribution to the model’s prediction of diabetes. Observing Fig 14, it is evident that an increase in value corresponds with an increase in SHAP values, signifying these features contribute positively to the likelihood of diabetes.

**Fig 15:**
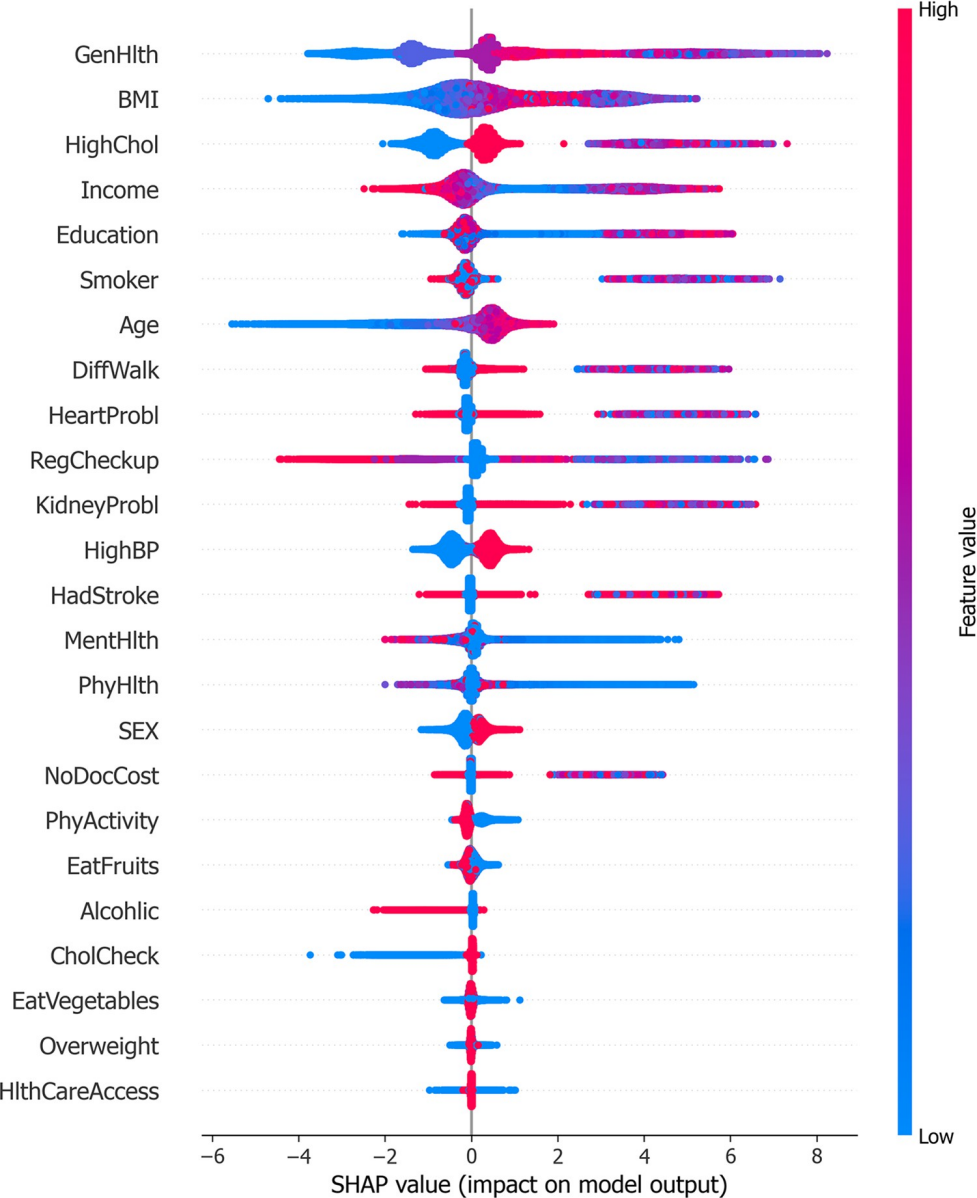
GA-XGBoost model SHAP feature density scatter plot.

**Fig 16:**
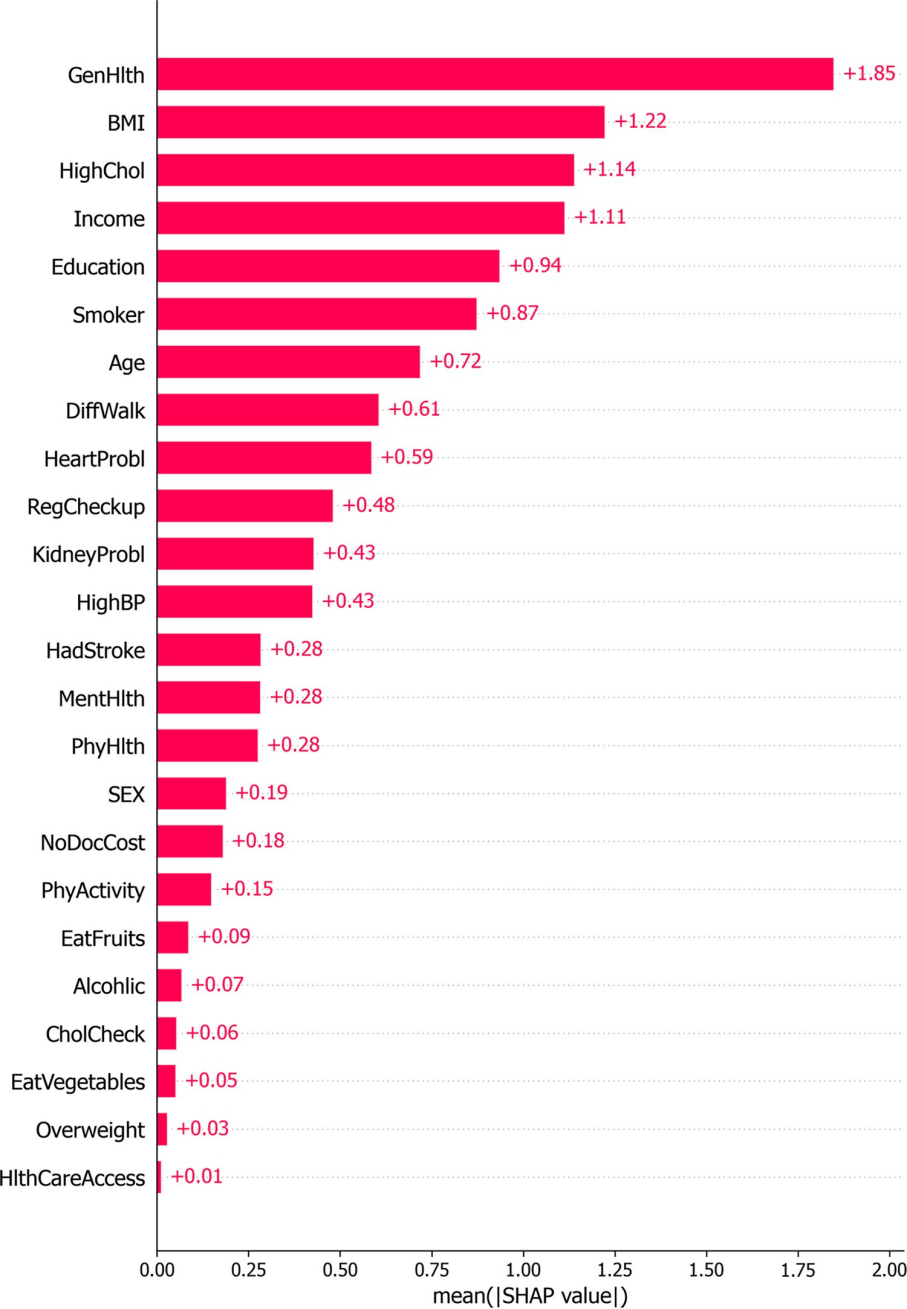
SHAP summary plot of feature importance ranking of GA-XGBoost model.

**Fig 17:**
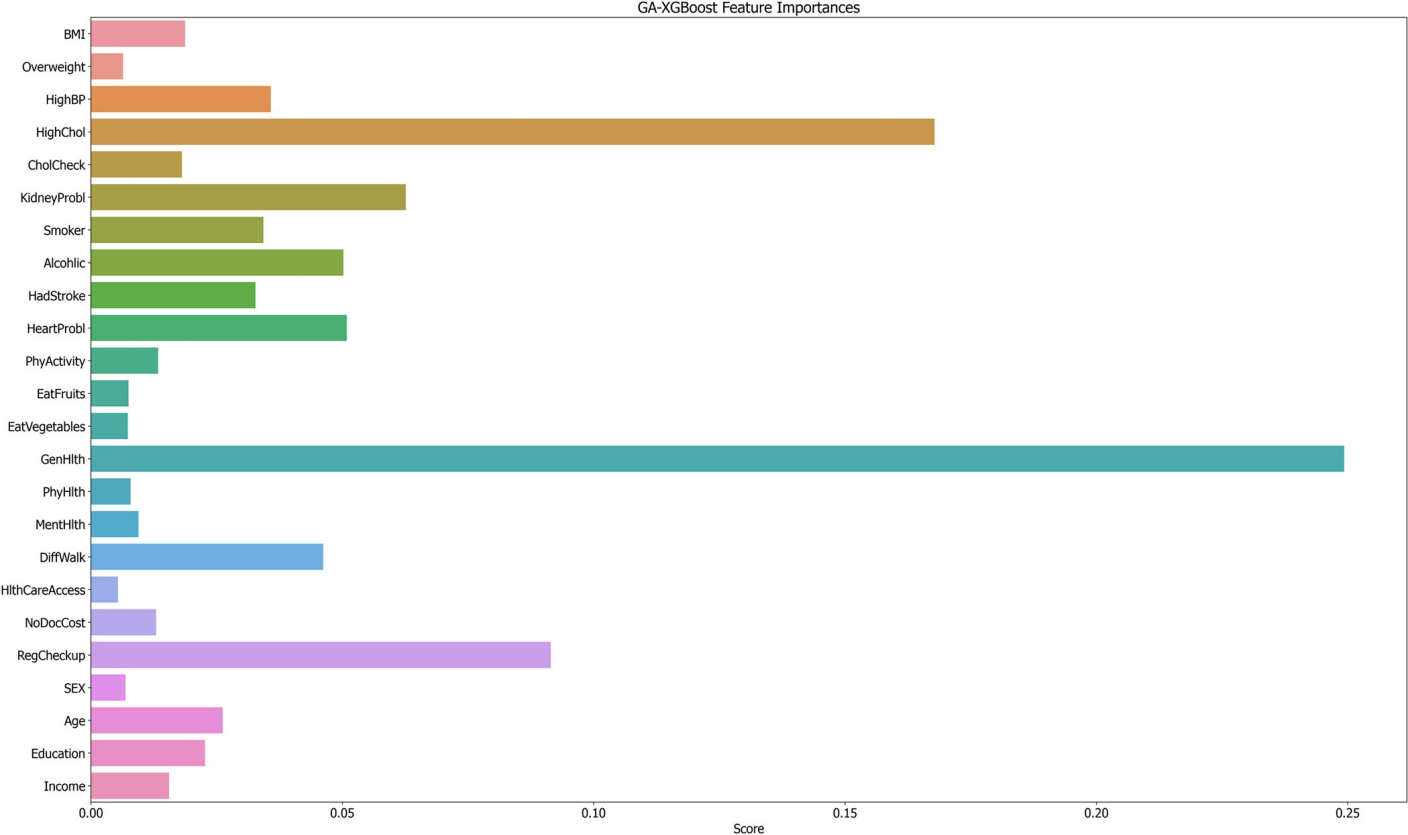
GA-XGBoost feature importances.

From Fig 14, it is evident that the predictions from the GA-XGBoost model exert a greater influence on the risk of developing diabetes compared to those from the LightGBM model. Therefore, an individual analysis is performed here specifically for the GA-XGBoost model. Figs 15 and 16 represent the SHAP feature density scatter plot and the SHAP feature importance ranking for the GA-XGBoost model, respectively. Fig 17 displays the ranking of feature importance for the GA-XGBoost model in terms of functionality.

In Fig 15, we notice that specific features, such as "GenHlth," "Age," "BMI," and "HighBP," significantly contribute positively to the model’s predictive results. This conclusion is drawn from the concentration of SHAP values for these features on the positive y-axis, signifying their affirmative correlation with the model’s forecasts. Specifically, an increment in the values of "GenHlth" and "HighChol" typically results in a higher predicted risk of diabetes by the model.

Conversely, features like " EatFruits " or "NoDocCost" may exert a negative influence on the model, given that their SHAP values are densely clustered on the negative y-axis. This implies that these elements could potentially decrease an individual’s likelihood of contracting diabetes. Additionally, Fig 16 illustrates the SHAP feature importance ranking for the GA-XGBoost model, which is an XGBoost model enhanced with a genetic algorithm. Fig 17 then presents the functional feature importance ranking for the identical model. Despite the clear visual disparities between the two rankings, they collectively emphasize the significance of "GenHlth," "HighChol," and "BMI" in evaluating an individual’s risk for diabetes. This reveals that, irrespective of the method employed for ranking feature importance, these three attributes are crucial for assessing diabetes risk. It is worth noting that "GenHlth" and "HighChol" are consistently recognized as the most impactful features regarding diabetes risk across both ranking methods, highlighting their critical role in diabetes risk prediction. This uniformity indicates that the prognostic significance of these features remains marked and consistent, irrespective of variations in the model’s internal architecture.

## 5 Discussion

Diabetes, as a worldwide health concern, necessitates early diagnosis for its prevention and management. However, current risk prediction models fall short in terms of accuracy and personalization. This research endeavored to enhance the precision and individualization of diabetes risk forecasting through sophisticated machine learning technologies. We discovered that the Stacking ensemble model surpasses other models across all assessed metrics, achieving an AUC of 98.90%, which is markedly higher than that of other individual models. The superior performance of the Stacking model is attributed to the collaborative effect of the GA-XGBoost algorithm and the LightGBM model. This integration not only bolsters the model’s predictive capabilities but also enhances its generalizability through feature selection. Compared to the traditional models referenced in literature [10], our Stacking model exhibits greater robustness when addressing imbalanced datasets. Moreover, our model has achieved interpretability breakthroughs by offering a deeper analysis of feature impacts through the SHAP framework. Through in-depth analysis of feature impact using the SHAP framework. The construction of this high-performance diabetes risk prediction model not only helps doctors to identify high-risk individuals early, improving the accuracy of diagnosis, but also promotes medical professionals to implement early intervention measures for high-risk groups, such as adjusting lifestyle and dietary habits, to prevent or delay the progression of diabetes. For chronic diseases like diabetes, which have no obvious early symptoms, such early identification and intervention are particularly important, helping doctors to formulate more personalized treatment plans for patients, thereby improving therapeutic effects. Although this study has achieved significant improvements in predictive accuracy, we also recognize that the limitations of the sample size may affect the model’s generalization ability. Future studies should validate on broader and more diverse datasets, and explore more model fusion strategies or apply deep learning techniques to diabetes risk prediction to further enhance the model’s predictive performance and interpretability. Overall, this study has successfully developed a high-performance diabetes risk prediction model and provided in-depth model interpretation through the SHAP framework. This not only helps patients better understand their health status and enhance self-management abilities, such as regularly monitoring blood sugar and actively participating in health management programs, but also integrates the model into clinical decision support systems, assisting doctors in making more scientific and rational decisions in the diagnosis and treatment process.

## 6 Conclusion

In the current research on diabetes risk prediction, traditional machine learning methods are primarily used but are plagued with issues such as low predictive accuracy and poor generalization capabilities. This paper constructs a Stacking model that integrates an XGBoost model optimized by a genetic algorithm and mixed sampling with LightGBM, and compares it with other single models through comparative experiments. Subsequently, to address the black box issue of ensemble models, this paper further employs an interpretable learning approach to elucidate the model’s predictive outcomes and utilizes the SHAP component for model explanation and analysis. Through experimentation, the following points have been concluded from this study.

1. To address the issue of imbalance in the predictive categories within the dataset, various sampling methods were employed to balance the dataset, with the mixed sampling approach proving to be the most effective. This aligns better with the actual imbalanced distribution of diabetes prevalence data.
2. Compared to traditional methods such as grid search, random search algorithms, and Bayesian optimization, hyperparameter tuning with a genetic algorithm more effectively enhanced the predictive accuracy of the XGBoost model, with all evaluation metrics showing varying degrees of improvement over the default parameter settings of the XGBoost model. The Stacking model, which integrates the improved XGBoost model with the LightGBM model, outperforms other traditional machine learning models across all evaluation metrics.
3. The SHAP visualization component can resolve the issue of ensemble learning not being able to predict the learning process, which is beneficial for understanding which features have a significant impact on the predictive outcomes.

Despite our model demonstrating high effectiveness in predicting the risk of diabetes, there is still room for improvement in feature selection and parameter optimization. To further enhance model performance, future research directions will focus on the following key points:

1. Optimization of feature selection methods: We will explore and compare different feature selection techniques, such as integrating PCA (Principal Component Analysis) methods, to identify and retain features that significantly impact the risk of diabetes while removing redundant or less influential features, thereby improving the model’s predictive accuracy and generalization capabilities.
2. Improvement of parameter optimization algorithms: Currently, the model’s parameter optimization process may be time-consuming. Therefore, we plan to adopt more efficient algorithms, such as PSO (Particle Swarm Optimization) algorithms, with the aim of finding optimal parameter combinations in less computing time, enhancing the model’s predictive performance.
3. Localization of the dataset: Given that the current research utilizes a dataset from the Kaggle platform, which may not fully reflect the actual situation of domestic patients, we plan to use datasets from domestic hospitals for model training and validation in future research to make the model more aligned with the characteristics and needs of domestic diabetes patients. Additionally, explore the application effects of the model in actual clinical environments.

Through these measures, we hope to construct a more accurate and efficient diabetes risk prediction model, providing stronger support for clinical decision-making and contributing to the prevention and management of diabetes on a global scale.
